# *Marinomonas mediterranea* synthesizes an R-type bacteriocin

**DOI:** 10.1128/aem.01273-23

**Published:** 2024-01-03

**Authors:** Patricia Lucas-Elío, Tarik ElAlami, Alicia Martínez, Antonio Sanchez-Amat

**Affiliations:** 1 Department of Genetics and Microbiology, University of Murcia, Murcia, Spain; Unversidad de los Andes, Bogotá, Colombia

**Keywords:** prophage, R-type bacteriocin, *Marinomonas*

## Abstract

Prophages integrated into bacterial genomes can become cryptic or defective prophages, which may evolve to provide various traits to bacterial cells. Previous research on *Marinomonas mediterranea* MMB-1 demonstrated the production of defective particles. In this study, an analysis of the genomes of three different strains (MMB-1, MMB-2, and MMB-3) revealed the presence of a region named MEDPRO1, spanning approximately 52 kb, coding for a defective prophage in strains MMB-1 and MMB-2. This prophage seems to have been lost in strain MMB-3, possibly due to the presence of spacers recognizing this region in an I-F CRISPR array in this strain. However, all three strains produce remarkably similar defective particles. Using strain MMB-1 as a model, mass spectrometry analyses indicated that the structural proteins of the defective particles are encoded by a second defective prophage situated within the MEDPRO2 region, spanning approximately 13 kb. This finding was further validated through the deletion of this second defective prophage. Genomic region analyses and the detection of antimicrobial activity of the defective prophage against other *Marinomonas* species suggest that it is an R-type bacteriocin. *Marinomonas mediterranea* synthesizes antimicrobial proteins with lysine oxidase activity, and the synthesis of an R-type bacteriocin constitutes an additional mechanism in microbial competition for the colonization of habitats such as the surface of marine plants.

**IMPORTANCE:**

The interactions between bacterial strains inhabiting the same environment determine the final composition of the microbiome. In this study, it is shown that some extracellular defective phage particles previously observed in *Marinomonas mediterranea* are in fact R-type bacteriocins showing antimicrobial activity against other *Marinomonas* strains. The operon coding for the R-type bacteriocin has been identified.

## INTRODUCTION

Prokaryotes have the ability to acquire and utilize exogenous genetic elements, such as phages, which can integrate into the bacterial genome as prophages. These prophages participate in defense mechanisms against invading elements or provide antimicrobial properties. Over time, they can evolve into cryptic or defective forms through a process called “domestication,” equipping cells with useful molecular mechanisms (1). Contractile injection systems (CIS) comprise a diverse group of nanomolecular structures, characterized by a rigid tube surrounded by a contractile sheath (2). Type VI secretion systems (T6SSs) are one type of CIS that span the cytoplasmic and outer membrane of Gram-negative bacteria. T6SSs deliver various effectors to both prokaryotic and eukaryotic target cells, including antibacterial proteins, toxins, and effectors that act on the cytoskeleton of eukaryotes (3). Extracellular CIS are secreted upon cell lysis (4) and have been utilized to deliver native or non-native payloads, such as CRISPR proteins, to various cell types. This utilization shows promising biotechnological applications (5).

Phage tail-like bacteriocins (PTLB), also known as tailocins (6), have been categorized into different types, including F-type, which is akin to flexible non-contractile *Siphoviridae* tails, R-type bacteriocins, which resemble the rigid contractile tails of *Myoviridae*, and other types of PTLB that exhibit similarities to various phages (7). Tailocins do not deliver any payload into their target cells but puncture their cell wall, destroying their membrane potential, which causes their lysis. R-type bacteriocins are only released upon cell lysis. The lysis of a few bacterial producer cells is believed to facilitate the utilization of R-type tailocins to benefit the colony as a whole in a competing scenario. The antimicrobial activity of R-type bacteriocins is exceptionally high, and it is estimated that a single R-bacteriocin is capable of lysing a cell (7). The genes coding for these bacteriocins are organized in clusters within the genome. These gene clusters encompass structural proteins, regulatory genes, and proteins involved in cell lysis and release of the bacteriocin (7). The cell lysis genes encode an endolysin and a holin, which work together to facilitate the access of the endolysin to the periplasmic space. The production of these macromolecules occurs within the producing cells, typically in response to DNA damage. Once released, the bacteriocins attach to the surface of receptor cells, utilizing the O-antigen of the lipopolysaccharide (LPS) as the receptor (8). It is generally considered that R-type bacteriocins exhibit intraspecific antimicrobial activity. However, it has also been observed that, in some cases, they can have an interspecific effect, targeting a broader range of species (9). The increase in antibiotic resistance among bacteria has sparked interest in alternative antimicrobial mechanisms, such as phages and bacteriocins, among others. Numerous studies have focused on R-type bacteriocins, particularly in plant-associated bacteria. These bacteriocins have been recognized as potential biocontrol agents against phytopathogens due to their high activity and specificity (10).

*Marinomonas mediterranea*, a marine Gram-negative bacterium, has served as a valuable model to study CRISPR-Cas systems. Previous studies have revealed that strain MMB-1 harbors two distinct types of CRISPR-Cas systems. One of these systems belongs to the I-F subtype, while the other belongs to the III-B subtype. The III-B system in MMB-1 has demonstrated the ability to acquire spacers from both RNA and DNA, facilitated by a fusion protein combining retrotranscriptase (RT) and Cas1 activities (11). Interestingly, different strains of *M. mediterranea* exhibit variations in their arrangement of CRISPR-Cas systems. For instance, strain MMB-2 (IVIA-Po-186) lacks a III-B system, while the III-B system in strain MMB-3 (CPR1) does not exhibit fusion with RT. Furthermore, there is limited conservation of spacers among the different strains (12).

In *M. mediterranea* MMB-1, the two CRISPR-Cas systems synergistically contribute to resistance against podoviruses (12). Notably, *M. mediterranea* MMB-1 is a lysogenic bacterium that releases defective prophages upon induction with mitomycin or UV, as confirmed by electron microscopy (13). However, the genetic components responsible for these prophages have not yet been identified at the genomic level. The aim of this study was to identify the genes encoding these defective particles, elucidate their biological function, and analyze their distribution across various *M. mediterranea* strains.

In this study, a region encoding a prophage of approximately 50 kb was identified in strains MMB-1 and MMB-2. Interestingly, the absence of this prophage in strain MMB-3 was found to be correlated with the presence of a CRISPR array containing spacers specifically targeting the missing prophage. This observation underscores the significant role that CRISPR-Cas systems have played in shaping the genomic diversity of *Marinomonas mediterranea*. In addition, it was observed that all three strains released defective particles upon induction with mitomycin. This study demonstrates that these particles are R-type bacteriocins encoded by a conserved 12-kb region present in the genomes of all analyzed strains.

Overall, these findings shed light on the intricate interplay between CRISPR-Cas systems, prophages, and the release of defective particles in *Marinomonas mediterranea*. The identification and characterization of these genetic elements provide valuable insights into their biological significance and potential biotechnological applications.

## MATERIALS AND METHODS

### Bacterial strains

Table 1 shows all the strains used in this study.

**TABLE 1:** Bacterial strains used in this study

Strain	Genotype/phenotype	References
*M. mediterranea* MMB-1	Wild-type strain	(14)
*M. mediterranea* MMB-1RSS	MMB-1 spontaneously Rif^r^	(12)
*M. mediterranea* MMB-1RSS ΔMEDPRO2	MMB-1RSS with a deletion of the MEDPRO2 region	This study
*M. mediterranea* MMB-2 (IVIA-Po-186)	Wild-type strain	(15)
*M. mediterranea* MMB-3 (CPR1)	Wild-type strain	(12)
*Marinomonas posidonica* (IVIA-Po-181)	Wild-type strain	(16)
*Marinomonas alcarazii* (IVIA-Po-14b)	Wild-type strain	(16)
*Marinomonas rhizomae* (IVIA-Po-145)	Wild-type strain	(16)
*Marinomonas foliarum* (IVIA-Po-155)	Wild-type strain	(16)
*Marinomonas aquiplantarum* (IVIA-Po-159)	Wild-type strain	(16)
*Marinomonas arctica* JCM 14976	Wild-type strain	(17)
*Marinomonas vaga* ATCC27119	Wild-type strain	(18)
*Marinomonas communis* ATCC27118	Wild-type strain	(18)
*Marinomonas dokdonensis* DSW10-10	Wild-type strain	(19)
*Marinomonas pontica* 46–16	Wild-type strain	(20)
*Marinomonas spartinae* SMJ19	Wild-type strain	(21)
*Marinomonas spartinae* SMJ28	Wild-type strain	(21)
*Marinomonas* sp. MWYL1	Wild-type strain	(22)

### Prophage detection in *M. mediterranea* genomes

The assembled genomes of strains MMB-1, MMB-2, and MMB-3 are deposited at NCBI under the genome assembly numbers ASM2847282v1 (CP047696.1) (23), ASM2847280v1 (CP047695.1), and ASM2847278v1 (CP047694.1) (12), respectively. The initial screening for prophage detection utilized several online servers, namely the PHASTER server (http://phaster.ca) (24), the Phage Hunter server (https://pro-hunter.genomics.cn/) (25), and the Prophinder server (http://aclame.ulb.ac.be/perl/Aclame/Prophages/prophinder.cgi) (26). The identified sequences detected, along with neighboring genes, were then subjected to manual inspection and analysis using BLAST (27). Additionally, the putative prophages identified in one genome were further examined in the other genomes.

### Purification of the released particles

The cultures of *M. mediterranea* growing in MMC2 medium (12) at 25°C until exponential phase (OD_600_ ≈ 0.3–0.4) were subjected to cellular lysis by the addition of mitomycin C at 0.1 µg/mL. After that, the cultures were centrifuged for 1 hour at 17,000 *× g*, and the supernatants were collected. The induced particles were then sedimented by ultracentrifugation at 58,000 *× g* for 1 hour at 4°C. The resulting pellet was later resuspended in SST (buffered saline solution) (14).

### Mass spectrometry (MS) of the released particles

The prophage suspensions were treated with ice-cold acetone (1:5, vol/vol) and centrifuged for 5 min at 14,000 × *g*. The resulting supernatant was discarded, and the pellet was allowed to dry. To reconstitute the pellet, it was dissolved in 50 mM ammonium bicarbonate pH 8.0 containing 0.01% ProteaseMax surfactant (Promega) at the original sample volume. The mixture was sonicated (Branson SLPe Digital Sonifier) for 10 seconds, with the amplitude set at 13%, factory-tuned frequency of 40 kHz, power output of up to 150 Watts, and a 0.125″ diameter tapered microtip, and the process was repeated five times.

Samples were subjected to protease digestion by using the ProteaseMax surfactant (Promega) and suspended at 0.01% in 50 mM ammonium bicarbonate, pH 8.5. Afterward, dithioerythritol was added and incubated at 56°C for 20 min. Alkylation was performed by adding 100 mM of iodoacetamide and incubating for 30 min at room temperature in the dark. The samples were then digested by adding 1 µg of Trypsin Gold Proteomics Grade (Promega) and incubating for 3 hours at 37°C. The reaction was halted by adding 0.1% formic acid. After digestion, the samples were filtered through a 0.2-µm membrane and dried using an Eppendorf Vacuum Concentrator model 5301.

Eventually, the samples were subjected to analysis by high-performance liquid chromatography-mass spectrometry following previously described protocols (28). The results obtained were checked with the protein sequences database for each genome.

### Antimicrobial activity

The antimicrobial activity of the induced particles was evaluated using an antibiogram test. One hundred microliters of exponential phase cultures of different *Marinomonas* strains were evenly distributed on MMC2 plates. Next, 10 µL drops of the induced cultures treated with mitomycin, or concentrated extracts obtained through centrifugation, were deposited on the bacterial lawn. The presence of antimicrobial activity was discerned by the formation of lysis halos within the bacterial lawns following an incubation period of 24–48 hours at 25°C.

### Deletion of the genomic region MEDPRO2

The *M. mediterranea* MMB-1RSS ΔMEDPRO2 deletion strain was generated using the unmarked counterselection strategy previously described (28). Flanking regions of MEDPRO2, adjacent to GV053_21210 and GV053_21300, were amplified and sequentially cloned into the suicide vector pEX18Gm. The primers used for the amplification of flanking fragments were MM5D (ACCGCTCTAGAaTtCGACTTTTACGC), MM5R (AAAAGTGGAGcTcGGCAGTGATGC), MM6D (TAACTGTgaGCTCGTAATTTGAGAGG), and MM6R (CAAGTTTAAGAAaGCTTTGATACAG), where the lowercase letters represent introduced mismatches for restriction cloning. The constructed vector was cloned into *Escherichia coli* S17-1 and mobilized by conjugation into the recipient strain. Following conjugation with MMB-1RSS, single recombination mutants were selected on MMCRif50Gm10 plates (29). Next, double recombination mutants were selected from MMC plates supplemented with 5% sucrose, and the deletion was confirmed by PCR.

### Electron microscopy

The concentrated induced cultures were visualized using electron microscopy at the Área Científico Técnica de Investigación of the University of Murcia. The samples were deposited onto carbon-coated grids and left to settle for 15 min. Finally, negative staining was performed by applying 20% uranyl acetate for 1 min. The measurement of the bacteriocin particles (SD ± SEM) was performed manually using Fiji (ImageJ 1.54) software in a total of 26 isolated and well-defined particles.

## RESULTS

### Detection of prophages in the genomes of *M. mediterranea* strains

In order to assess the presence of prophages in the genomes of three distinct *Marinomonas mediterranea* strains, the software tools Phage Hunter (25), PHASTER (24), and Prophinder (26) were employed. The identified sequences and their genomic regions, as indicated by the aforementioned programs, were manually inspected and annotated. Several phage-related genes were found in two distinct genomic regions that were named MEDPRO1 and MEDPRO2. The genomes were analyzed for the presence of other genomic regions encoding potential phage proteins, and only two genes (GV053_3670 and GV053_3671 in strain MMB-1) encoding putative tail fiber proteins were conserved in all three strains. These two genes were not further considered in this study due to the absence of other phage-related genes associated with them.

#### Genomic region MEDPRO1

In the MMB-1 strain, the Phage Hunter program identified an “ambiguous” prophage region spanning from gene GV053_09220 to GV053_09450. Additionally, PHASTER detected a putative smaller prophage region extending from GV053_09230 to GV053_09415, followed by additional DNA corresponding to an *attL* region. Manual analysis of the genomic surroundings of these regions revealed the presence of phage-related genes, raising questions about the size of the prophage. To define the boundaries of this region, its presence in other *Marinomonas mediterranea* strains was investigated. It was found that the genes upstream of GV053_09140 and downstream of GV053_09460 were conserved in *M. mediterranea* MMB-2 and MMB-3, and were not related to phages. However, the three strains showed differences in the region between the conserved genes (Fig. S1). This region, termed MEDPRO-1, which covers approximately 52 kb from GV053_09140 to GV053_09460 in MMB-1, underwent detailed analysis, with gene annotations manually verified (Table S1). The regions in strains MMB-1 and MMB-2 exhibited a similar genomic arrangement with a high degree of synteny (Fig. S1). BLAST analysis of the proteins encoded in the region MEDPRO1 revealed similarity to proteins of the *Vibrio* phage VP882. Those that gave, in a protein BLAST, an expected value below 1E-10 are marked in red in Fig. S1. MEDPRO-1 appears to result from the integration of part of a VP882-related phage into an *M. mediterranea* strain. Evolution led to the acquisition of additional mobile genetic elements (MGEs), resulting in defective prophages. Strain MMB-3 had only a few remaining genes in the MEDPRO1 region, suggesting a lost prophage. CRISPR-Cas systems may have played a role in its absence, as some spacers matched prophage-related genes in MMB-3. It was observed that the second spacer in array 8 of the I-F system of strain MMB-3 perfectly matches the genes GV053_09365 in MMB-1 and GV054_09205 in MMB-2, which encode tail proteins. Additionally, the third spacer in the same array exhibits three mismatches with the genes GV053_09355 and GV054_09195. These findings suggest that a prophage similar to those found in strains MMB-1 and MMB-2 was eliminated from the genome of *M. mediterranea* MMB-3 after the acquisition of these spacers.

#### Genomic region MEDPRO2

**Fig 1:**
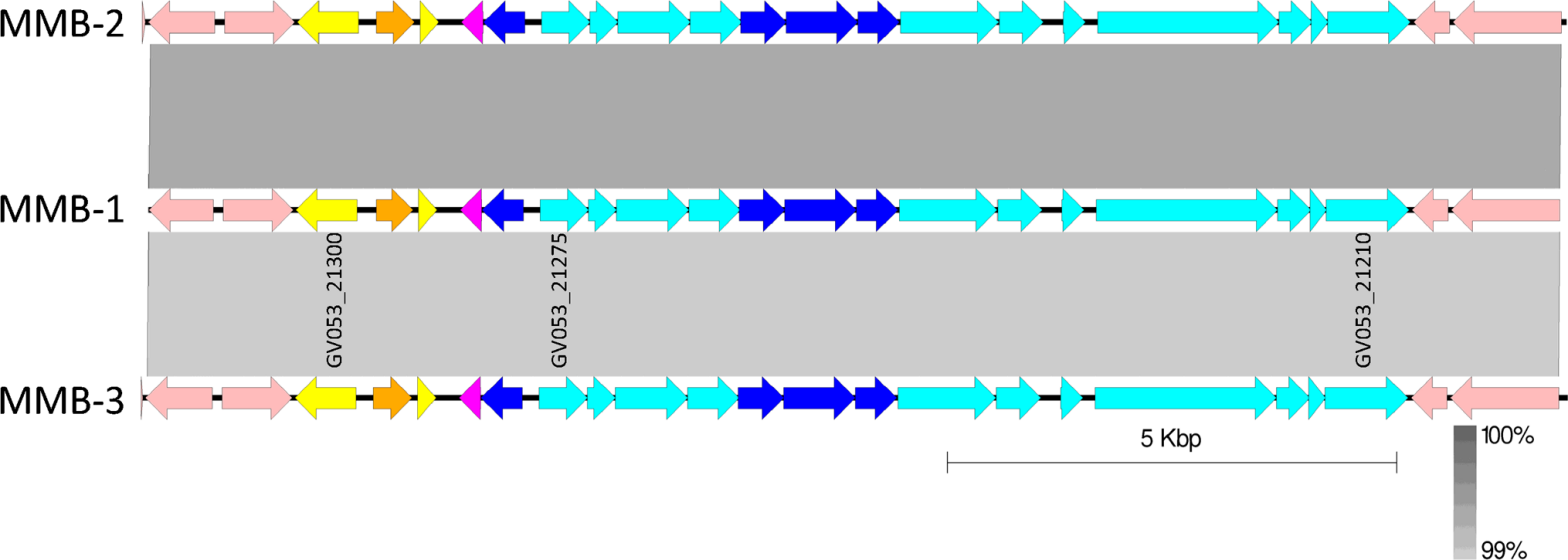
Genomic synteny between the MEDPRO2 regions in three *M. mediterranea* strains. Arrows represent the locations of coding sequences and shaded gray lines the homology between them. Genes encoding non-prophage proteins are in pink. Colors in genes mark specific functions of the encoded proteins: phage structural proteins are in cyan, regulatory genes are in yellow, putative holins are in magenta, lysozymes in orange, and other prophage proteins are in dark blue. The blastn genome comparison was performed and visualized with Easyfig 2.2.5.

In strain MMB-1, a potential prophage was identified using Prophinder (26). It spans approximately 12 kb from genes GV053_21210 to GV053_21300. Interestingly, a similar prophage located in the exact same genomic region and exhibiting a similarity higher than 99% at the DNA level was detected in the other two analyzed *M. mediterranea* strains, MMB-2 and MMB-3 (Fig. 1).

The annotation of this putative prophage was further investigated using blastp against multiple databases to identify proteins with the highest similarity. The genomic organization of the prophage, named MEDPRO2, bears resemblance to that of tail-like bacteriocins, particularly type-R bacteriocins (7). The downstream region contains genes encoding structural proteins, while the upstream region harbors genes encoding regulatory proteins (Fig. 1; Table S2). Additionally, in strain MMB-1, there is a gene, GV053_21295, encoding a lysozyme that belongs to the cl00222: Lyz-like Superfamily.

Further analysis of the products encoded by genes GV053_21285 and GV053_21280 using the DeepTMHMM program (30) revealed that both genes code for small proteins of 80 and 153 amino acids, respectively, with transmembrane domains. The product of GV053_21285 is a strong candidate for being a holin due to its small size of 80 amino acids, possession of a single transmembrane domain, and a highly hydrophilic, charged C-terminal region enriched with acidic residues. These characteristics align with the general attributes of holins (31). The remaining 14 genes encode various structural proteins associated with the tail and base plate. Notably, no genes encoding homologs of proteins involved in DNA replication or capsid formation have been detected.

### Induction of prophages in the three *M. mediterranea* strains

**Fig 2:**
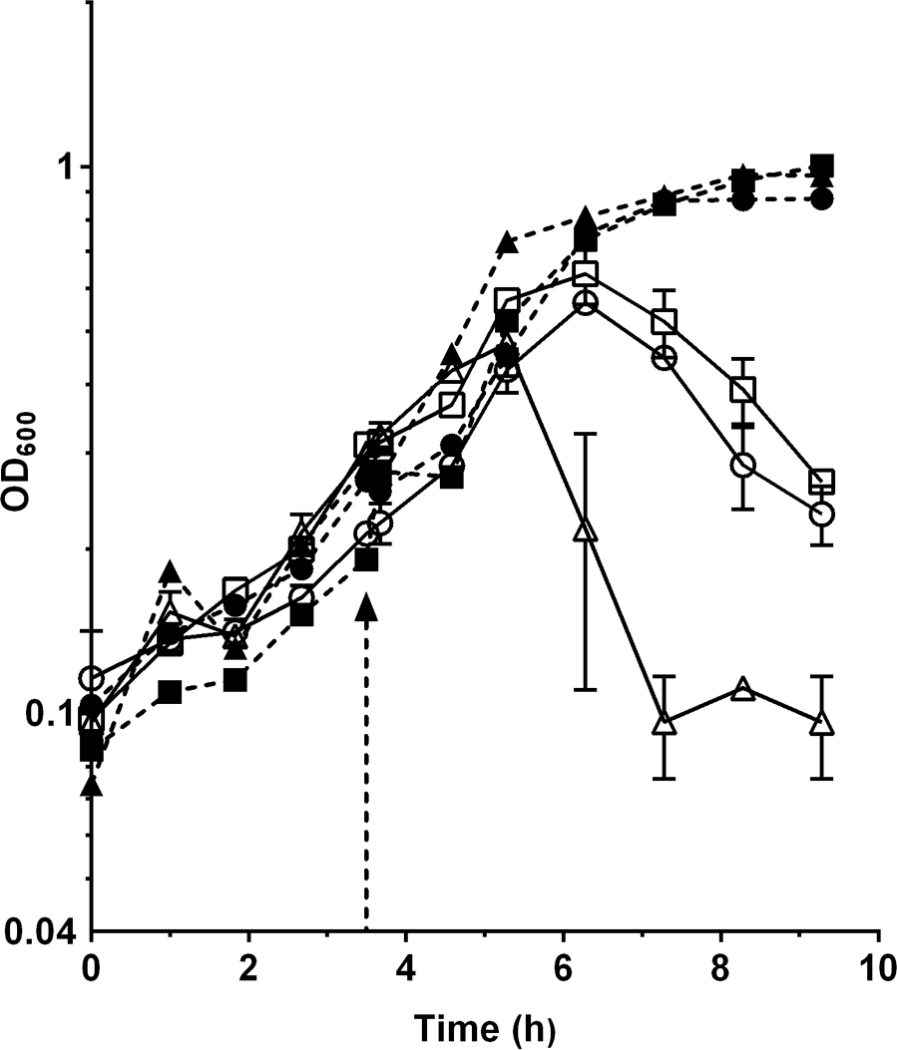
Induction by mitomycin of prophages in MMC2 medium. Evolution of OD_600_ of *M. mediterranea* cultures of strains MMB-1 (circles), MMB-2 (squares), and MMB-3 (triangles). Closed symbols represent control cultures, while open ones are cultures induced by mitomycin at the time indicated by the arrow.

Mitomycin C is widely recognized as a potent inducer of prophages in bacterial cultures. Treatment with mitomycin C can lead to the activation of prophages integrated within the bacterial genome which leads to cell lysis. In this study, the treatment with mitomycin C resulted in the lysis of cultures of all three tested *M. mediterranea* strains (Fig. 2). This lysis event is likely associated with the induction of prophages and the subsequent release of phage particles. It is worth mentioning that in strain MMB-3, which exclusively harbors the prophage MEDPRO2, the lysis phenomenon is particularly pronounced and occurs at an earlier stage. Additionally, exposure of the cultures to UV radiation also induced cell lysis (data not shown).

### Microscopic observation of the induced cultures

**Fig 3:**
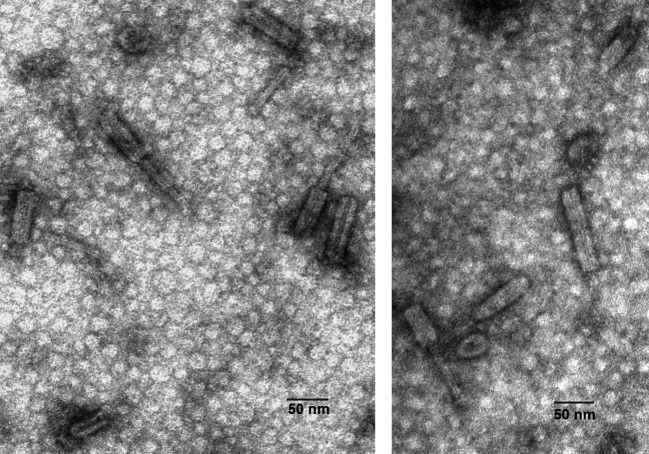
Transmission electron micrograph of defective phage particles stained with uranyl acetate released after mitomycin induction in cultures of strain MMB-2 (left) and MMB-3 (right).

Microscopic examination of the induced cultures revealed the presence of similar particles in all three analyzed strains, closely resembling the particles previously described in strain MMB-1 (Fig. 3) (13). The particles observed bear similarity to R-type bacteriocins, the most abundant particles in the micrographs were complete contracted forms, although a few empty sheaths and complete particles, mostly aggregated, were also observed. The average particle length measured 153.29 ± 4.77 nm, with the contracted sheath dimensions of 72.16 ± 2.38 nm in length and 21.20 ± 0.44 nm in width. The tube dimensions were recorded as 81.15 ± 4.18 nm in length and 8.67 ± 0.33 nm in width. In strain MMB-3 where the MEDPRO1 region is absent, the observation of these phage particles suggests that they are encoded by genes within the MEDPRO2 region.

### Purification of the defective phages and MS analysis of the supernatants

The supernatants of the induced cultures were concentrated and purified according to the description in the Materials and Methods section. While multiple peptides were detected, only proteins from the MEDPRO2 region, but not from MEDPRO1, were identified (Table 2). Curiously, strain MMB-3 showed the lowest abundance of detected peptides and types of proteins, despite exhibiting pronounced lysis of the culture following induction. One possible explanation for this observation could be the high abundance of ribosomal peptides detected in this sample, whose signals may interfere with the detection of phage peptides. Interestingly, the most abundant proteins in R-bacteriocins, specifically those constituting the tail tube and the tail sheath (32), were detected in all three strains after mitomycin induction (Table 2). The third protein found in all the supernatants (encoded by GV053_21250 in MMB-1) encodes a hypothetical protein, although the analysis of its sequence in the PredictProtein server (https://predictprotein.org/) revealed similarities with automatically annotated phage tail proteins in the genera *Vibrio* and *Chromobacterium,* which would be compatible with its detection in the released particles. Notably, in the induced cultures of strain MMB-1, the prophage peptides detected correspond to gene products serving as structural proteins (GV053_21220 and GV053_21260 as phage tail proteins; GV053_21275 and GV053_21265 as baseplate assembly proteins; and homologs of GV053_21225 appear to dictate tail length). The sole exception is the detection of a protein belonging to the late control D family group (GV053_21210), involved in cell lysis and virion maturation. Proteins encoded from GV053_21300 to GV053_21280 do not seem to be part of the defective particle, as they were not detected in mass spectrometry analyses. This finding aligns with their predicted roles as regulatory proteins (GV053_21300 and GV053_21290), lysozyme (GV053_21295), and a holin (GV053_21280), essential for the release of the particle.

**TABLE 2:** Identification of prophage-related peptides in the supernatants of *M. mediterranea* cultures induced with mitomycin C, using mass spectrometry^*a*^

MMB-1 locus-tag	Gene product	MMB-1	MMB-2	MMB-3
GV053_21275	Phage baseplate assembly protein V	2 (23)	1 (8)	–^*b*^
GV053_21265	Baseplate assembly J/gp47 family protein	10 (31)	1 (5)	–
GV053_21260	Phage tail protein	7 (28)	–	–
GV053_21255	Hypothetical protein	13 (34)	10 (15)	–
GV053_21250	Hypothetical protein	21 (45)	35 (44)	3 (24)
GV053_21240	Tail sheath protein	23 (40)	60 (34)	5 (5)
GV053_21235	Phage major tail tube protein	37 (73)	23 (40)	14 (26)
GV053_21225	Tail length tape measure protein	14 (26)	–	–
GV053_21220	Phage tail protein	2 (30)	–	–
**GV053_21210**	Late control D family protein	4 (11)	–	–

^*a*^ The numbers indicate the total count of detected peptides, while the numbers in parentheses represent the percentage of amino acids from each protein that was detected in the analysis.

^*b*^ –, no peptide was detected.

### Confirmation of the identity of the genes coding for the defective prophage by gene deletion

In order to further confirm that the lysis of the induced cultures is specifically the result of the activation of MEDPRO2, a strain MMB-1RSS ΔMEDPRO2 was generated by deleting the genes encoding that region. It was observed that under the conditions of the experiment, no lysis was induced by mitomycin in the strain with the deletion (Fig. S2). Furthermore, it was noted that in the genetic background of strain MMB-1RSS, the induction by mitomycin was not as evident as pronounced as in strain MMB-1, possibly indicating changes in this strain, which was selected as a spontaneous rifampicin-resistant mutant. Even under these conditions, phage particles were observed in the supernatants of the induced cultures of strain MMB-1RSS. In contrast, no phage particles were observed in the case of the strain ΔMEDPRO2.

### Antimicrobial activity of the phage particles

The findings presented in the preceding sections suggest that the genomic region here named MEDPRO2, which is present in all three *M. mediterranea* strains analyzed, may encode an R-type bacteriocin. Initial experiments conducted with the three wild-type strains demonstrated antimicrobial activity against *Marinomonas* sp. MWYL1 in the culture supernatants following mitomycin treatment. Subsequently, the bactericidal activity of the culture supernatants from strains MMB-1RSS and MMB-1RSS ΔMEDPRO2 was tested against various *Marinomonas* strains. The results revealed that only two strains, *M. aquiplantarum* and *Marinomonas* sp. MWYL1, were susceptible to the product encoded in the MEDPRO2 region confirming that it encodes an R-type bacteriocin (Table 3).

**TABLE 3:** Antimicrobial activity of supernatants of mitomycin-induced cultures of strains MMB-1 and MMB-1RSS ΔMEDPRO2

	MMB-1RSS	MMB-1RSS ΔMEDPRO2
*Marinomonas posidonica*	R	R
*Marinomonas arctica*	R	R
*Marinomonas vaga*	R	R
*Marinomonas communis*	R	R
*Marinomonas dokdonensis*	R	R
*Marinomonas pontica*	R	R
*Marinomonas spartinae* SMJ19	R	R
*Marinomonas spartinae* SMJ28	R	R
*Marinomonas alcarazii*	R	R
*Marinomonas rhizomae*	R	R
*Marinomonas foliarum*	R	R
*Marinomonas aquiplantarum*	S	R
*Marinomonas mediterranea* MMB-1RSS	R	R
*M. mediterranea* MMB-1RSS ΔMEDPRO2	R	R
*Marinomonas* MWYL1	S	R
*Marinomonas mediterranea* MMB-3	R	R
*Marinomonas mediterranea* MMB-2	R	R

## DISCUSSION

The evolution of bacterial genomes is significantly influenced by the gain or loss of MGEs, such as plasmids or prophages. The presence of CRISPR-Cas systems can serve as a barrier against the acquisition of MGEs. For instance, studies conducted on *Pseudomonas aeruginosa* have demonstrated a decrease in the number of prophages and integrative conjugative elements in genomes containing active CRISPR-Cas systems (33). Similarly, other studies have shown a negative correlation between the presence of CRISPR-Cas systems and plasmids acquisition, although no evidence was found in the case of prophages (34).

*Marinomonas mediterranea* is a Gram-negative marine bacterial species, with three strains described so far that contain different arrangements of CRISPR-Cas systems (12). In the strain MMB-1, it has been observed that certain spacers in the I-F system can be utilized by both the I-F and III-B CRISPR-Cas systems, providing resistance against podoviruses (12). No other targets have been identified for the remaining spacers in these strains.

A previous study observed the release of defective phage particles in the supernatants of mitomycin-induced cultures of strain MMB-1 (13). In this study, using various programs, we identified two genomic regions enriched in phage genes that potentially encode prophages. One of these regions, named MEDPRO1, is approximately 52 kb in size and is detected in strains MMB-1 and MMB-2. In this region, most of the genes coding for the tail and baseplate proteins are similar to those found in *Vibrio* phage VP882. However, the genes coding for the phage terminase large subunit, portal protein, head decoration protein, and major capsid are similar to the genes in other phages. This could be the result of the integration of a hybrid phage, but the presence of several integrase genes suggests that this region may have undergone the integration of multiple prophages, similar to observations in other bacteria (35). In strain MMB-3, where this prophage has not been detected, two spacers match genes coding for phage tail proteins. This finding could explain the absence of that particular prophage since, in CRISPR-Cas systems, the presence of spacers matching prophages is generally incompatible with the presence of those prophages (36, 37). The presence of a gene (GV055_09280) in the prophage in the other two *Marinomonas* strains suggests that the acquisition of the spacers occurred after prophage integration and that the phage was nearly completely eliminated by the activity of the CRISPR-Cas system.

Multiple lines of evidence indicate that the defective particles detected in the supernatants are encoded by genes in MEDPRO2. First, they are observed in the supernatants of induced cultures of all three analyzed strains, so the genes coding for them must be present in all the strains as is the case of the MEDPRO2 region. Second, mass spectrometry analyses identified products from that genomic region, while no products were found from MEDPRO1. Third, in the strain with the deletion of MEDPRO2, mitomycin does not induce cell lysis or the release of defective particles. The genomic organization of MEDPRO2 and its antimicrobial activity against two different *Marinomonas* strains support that MEDPRO2 encodes an R-type bacteriocin.

R-type bacteriocins are synthesized by various Gram-negative bacteria. So far, there is only a description of R-type bacteriocins from Gram-positive bacteria, the diffocin synthesized by *Clostridium difficile* (7, 32). R-type bacteriocins from *Pseudomonas aeruginosa* are being extensively studied due to their potential as antimicrobial agents (38). One of the first R-type bacteriocins was described in a marine *Vibrio* (39).

A BLAST analysis was conducted using the protein sequences encoded by the *M. mediterranea* R-type bacteriocin against sequences of other R-type bacteriocins, including fluorescin (40), syringacin (40), R bacteriocin of *Pseudomonas aeruginosa* PAO1 (7), R-type bacteriocins of *Pseudomonas chlororaphis* (41), xenorhabdicin (42), carotovorocin (43), and diffocin (32). The results revealed that the highest number of proteins detected as similar to MEDPRO2 were those of the R2-type bacteriocin of *P. aeruginosa*. The most easily detected proteins in the mass spectrometry analyses were those coding for the sheath protein and the tail tube. In both cases, the most similar proteins in the BLAST analyses were from the R2-type bacteriocin synthesized by *P. aeruginosa* PAO1. For instance, the sheath proteins of *M. mediterranea* MMB-1 and *P. aeruginosa* PAO1 showed 26.2% identity and 42.3% similarity, while the tail tube proteins showed 29.1% identity and 62.4% similarity. In terms of size, the R-type bacteriocin in *M. mediterranea* measures 153.29 ± 4.77 nm, which falls within the middle range compared to other bacteriocins that typically range from 80 to 184 nm (44). The most similar size is exhibited by the largest bacteriocin found in *Pseudomonas chlororaphis*, with a length of 149 ± 0.11 nm (41).

In the field of aquaculture, the study of R-type bacteriocins and other types of antimicrobial compounds synthesized by marine bacteria is of interest, especially considering the emergence of antibiotic resistance in pathogenic bacteria (45). In this context, the characterization of a bacteriocin synthesized by *M. mediterranea* holds significant relevance. Furthermore, this microorganism has been found to produce an antimicrobial protein with lysine oxidase activity, which has been implicated in biofilm formation and colonization of surfaces (46). The presence of this enzymatic activity, along with the production of the R-type bacteriocin described in this paper, may contribute to the antimicrobial properties and colonization potential of *M. mediterranea*. Understanding the mechanisms underlying these antimicrobial activities can provide valuable insights for addressing bacterial infections in aquaculture systems, where biofilm formation and surface colonization are common challenges.

## Data Availability

The assembled genomes of strains MMB-1, MMB-2, and MMB-3 are deposited at NCBI under the genome assembly numbers ASM2847282v1 (CP047696.1), ASM2847280v1 (CP047695.1), and ASM2847278v1 (CP047694.1), respectively.

## SUPPLEMENTAL MATERIAL

Tables S1 and S2 aem.01273-23-s0001.docx Genes in genomic regions MEDPRO1 and MEDPRO2

Fig. S1 and S2 aem.01273-23-s0002.pdf Supplementary figures.

ASM does not own the copyrights to Supplemental Material that may be linked to, or accessed through, an article. The authors have granted ASM a non-exclusive, world-wide license to publish the Supplemental Material files. Please contact the corresponding author directly for reuse.
