# Supplementary material for: Marinomonas mediterranea synthesizes an R-type bacteriocin
Source: Appl Environ Microbiol. 2024 Jan 3;90(1):e01273-23. doi: 10.1128/aem.01273-23 (PMC10870725; doi:10.1128/aem.01273-23)
Supplement: Tables S1 and S2 — Genes in genomic regions MEDPRO1 and MEDPRO2 [file aem.01273-23-s0001.docx]

**Table S1. Genes in the MEDPRO1 region in *M. mediterranea* strains.**

The locus tag in the different genomes is indicated. Locus tag in blue indicate high similarity to genes in Vibrio phage VP882. The asteriks indicate targeting by spacers in the I-F system of strain MMB-3.

*GV053_09355. Protospacer from position: 2031902-2031933 with 3 mismatches with spacer_IF_8_3 (I_F System array 8 spacer 3)

**GV054_09195. Protospacer from 1986149-1986180 with three mismatches with spacer MMB-3_IF8_3

*** GV053_09365. Protospacer from position 2033530-2033499 with 100% identity in reverse orientation with spacer MMB-3_IF8_2 (I_F System array 8 spacer 2)

****GV054_09205. Protospacer from position: 1987777-1987746 with 100% identity in reverse orientation with spacer MMB-3_IF8_2

| **MMB-1 Locus-tag** | **MMB-2 Locus tag** | **MMB-3 Locus tag** | **Gene product** |
| --- | --- | --- | --- |
| GV053_09140 | GV054_08975 | absent | Tyrosine-type recombinase/integrase |
| GV053_09145 | GV054_08980 | absent | Pyocin activator PrtN family protein |
|  |  | absent |  |
| GV053_09150 | GV054_08985 | absent | Hyp |
| GV053_09155 | GV054_08990 | absent | Hyp |
| GV053_09160 | GV054_08995 | absent | CsrA |
| GV053_09165 | GV054_09000 | absent | ImmA/IrrE family metallo-endopeptidase |
| GV053_09170 | GV054_09005 | absent | DUF4411 family protein |
| GV053_09175 | GV054_09010 | absent | Helix-turn-helix domain-containing protein |
| GV053_09180 | GV054_09015 | absent | Helix-turn-helix domain-containing protein |
| GV053_09185 | GV054_09020 | absent | Phage regulatory CII family protein |
| GV053_09190 | GV054_09025 | absent | DUF2786 domain-containing protein |
| GV053_09195 | GV054_09030 | absent | Phage regulatory CII family protein |
| GV053_09200 | GV054_09035 | absent | Hyp |
| GV053_09205 | GV054_09040 | absent | Hyp |
| GV053_09210 | GV054_09045 | absent | Hyp |
| GV053_09215 | GV054_09050 | absent | Tyrosine-type recombinase/integrase |
| GV053_09220 | GV054_09055 | absent | Hyp |
| GV053_09225 | GV054_09060 | absent | Hyp |
| GV053_09230 | GV054_09065 | absent | KilA-N domain-containing protein |
| absent | GV054_09070 | absent | Hyp |
| GV053_09235 | GV054_09075 | absent | Translesion error-prone DNA polymerase V autoproteolytic subunit UmuD |
| GV053_09240 | GV054_09080 | absent | Translesion error-prone DNA polymerase V subunit UmuCumuC |
|  |  | absent |  |
| GV053_09245 | GV054_09085 | absent | Hyp |
| GV053_09250 | GV054_09090 | absent | Holin |
| GV053_09255 | GV054_09095 | absent | Hyp |
| GV053_09260 | GV054_09100 | absent | Putative terminase small subunit |
| GV053_09265 | GV054_09105 | absent | Phage terminase large subunit family protein |
| GV053_09270 | GV054_09110 | absent | Hyp |
| **GV053_09275** | **GV054_09115** | absent | **Phage portal protein** |
| GV053_09280 | GV054_09120 | absent | ATP-dependent Clp protease proteolytic subunit ClpP |
| **GV053_09285** | **GV054_09125** | absent | **Head decoration protein** |
| **GV053_09290** | **GV054_09130** | absent | **Major capsid protein** |
| GV053_09295 | GV054_09135 | absent | Hyp |
| **GV053_09300** | **GV054_09140** | absent | **Tail completion protein** |
| GV053_09305 | GV054_09145 | absent | Hyp |
| **GV053_09310** | **GV054_09150** | absent | **Phage baseplate assembly protein V** |
| GV053_09315 | GV054_09155 | absent | Hyp |
| **GV053_09320** | **GV054_09160** | absent | **Phage baseplate protein** |
| **GV053_09325** | **GV054_09165** | absent | **Baseplate assembly protein** |
| **GV053_09330** | **GV054_09170** | absent | **Phage tail protein I** |
| **GV053_09335** | **GV054_09175** | absent | **Phage tail fiber protein** |
| GV053_09340 | GV054_09180 | absent | Hyp |
| GV053_09345 | GV054_09185 | absent | Hyp |
| GV053_09350 | GV054_09190 | absent | Hyp |
| **GV053_09355*** | **GV054_09195**** | absent | **Phage tail protein ó phage sheath protein** |
| **GV053_09360** | **GV054_09200** | absent | **Phage major tail tube protein** |
| **GV053_09365***** | **GV054_09205****** | absent | **Phage tail assembly protein** |
| **GV053_09370** | **GV054_09210** | absent | **Phage tail tape measure protein** |
| absent | GV054_09215 | absent | Phage antirepressor KilAC domain-containing protein |
| absent | GV054_09220 | absent | TIR domain-containing protein |
| absent | GV054_09225 | absent | Hyp |
| absent | GV054_09230 | absent | Transcriptional regulator |
| absent | absent | GV055_09270 | Hyp |
| absent | GV054_09235 | absent | Hyp |
| absent | GV054_09240 | absent | Hyp |
| GV053_09375 | absent | absent | IS30 family transposase ¿RNAse H? |
| GV053_09380 | absent | absent | Hyp |
| GV053_09385 | absent | absent | Hyp |
| **GV053_09390** | **GV054_09245** | **GV055_09280** | **phage tail protein** |
| **GV053_09395** | **GV054_09250** | **GV055_09285** | **phage tail protein ó tail X ó baeplate hub** |
| **GV053_09400** | **GV054_09255** | absent | **phage late control D family protein ó contractile injection system protein, VgrG/Pvc8 family ó Tail protein** |
| absent | GV054_09260 | absent | Hyp |
| absent | GV054_09265 | absent | Hyp |
| GV053_09405 | absent | absent | Hyp |
| GV053_09410 | absent | absent | Hyp |
| GV053_09415 | absent | absent | IS30 family transposase |
| GV053_09430 | GV054_09270 | absent | KilA-N domain-containing protein |
| absent | GV054_09275 | absent | Hyp |
| GV053_09435 | absent | absent | Endonuclease I |
| absent | absent | GV055_09245 | IS256 family transposase |
| absent | absent | GV055_09250 | TnpB |
| GV053_09440 | absent | GV055_09255 | Hyp |
| GV053_09445 | absent | GV055_09260 | Alpha/beta fold hydrolase |
| GV053_09450 | absent | GV055_09265 | Helix-turn-helix transcriptional regulator |
| absent | absent | GV055_09275 | Hyp |
| GV053_09455 | GV054_09280 |  | Tyrosine-type recombinase/integrase |
| GV053_09460 | GV054_09285 |  | Hyp |

**Table S2. Genes in prophages MEDPRO2 in *M. mediterranea* MMB-1, MMB-2 and MMB-3.**

| **MMB-1 locus tag** | **MMB-2 locus tag** | **MMB-3 locus tag** | **Gene product** | |
| --- | --- | --- | --- | --- |
| GV053_21300 | GV054_21270 | GV055_21340 | XRE family transcriptional regulator | |
| GV053_21295 | GV054_21265 | GV055_21335 | Lysozyme |  |
| GV053_21290 | GV054_21260 | GV055_21330 | Transcriptional regulator NrdR | |
| GV053_21285 | GV054_21255 | GV055_21325 | Holin |  |
| GV053_21280 | GV054_21250 | GV055_21320 | Hypothetical protein | |
| GV053_21275 | GV054_21245 | GV055_21315 | Phage baseplate assembly protein V | |
| GV053_21270 | GV054_21240 | GV055_21310 | Baseplate assembly GPW/gp25 family protein | |
| GV053_21265 | GV054_21235 | GV055_21305 | Baseplate assembly J/gp47 family protein | |
| GV053_21260 | GV054_21230 | GV055_21300 | Phage tail protein | |
| GV053_21255 | GV054_21225 | GV055_21295 | Hypothetical protein | |
| GV053_21250 | GV054_21220 | GV055_21290 | Hypothetical protein | |
| GV053_21245 | GV054_21215 | GV055_21285 | Hypothetical protein | |
| GV053_21240 | GV054_21210 | GV055_21280 | Tail sheath protein | |
| GV053_21235 | GV054_21205 | GV055_21275 | Phage major tail tube protein | |
| GV053_21230 | GV054_21200 | GV055_21270 | Phage tail assembly protein | |
| GV053_21225 | GV054_21195 | GV055_21265 | Tail length tape measure protein | |
| GV053_21220 | GV054_21190 | GV055_21260 | Phage tail protein | |
| GV053_21215 | GV054_21185 | GV055_21255 | Phage tail protein | |
| GV053_21210 | GV054_21180 | GV055_21250 | Late control D family protein | |
