# Supplementary material for: Marinomonas mediterranea synthesizes an R-type bacteriocin
Source: Appl Environ Microbiol. 2024 Jan 3;90(1):e01273-23. doi: 10.1128/aem.01273-23 (PMC10870725; doi:10.1128/aem.01273-23)
Supplement: Fig. S1 and S2 — Supplementary figures. [file aem.01273-23-s0002.pdf]

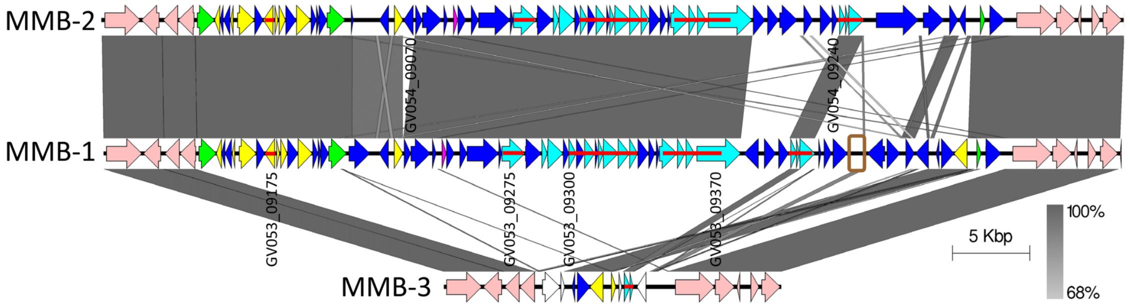

Figure S1. Genomic synteny between the MEDPRO1 regions in three *M. mediterranea* strains. Genes encoding non-prophage proteins are in pink. In strain MMB-1 these are genes upstream GV053_09140 and dowstream GV053_09460. Arrows represent the locations of coding sequences and shaded grey lines the homology between them. Colours in genes mark specific functions of the encoded proteins: phage structural proteins in cyan, regulatory proteins in yellow, integrases in green, holins are in magenta, other prophage proteins are in dark blue or white (in MMB-3 lacking the prophage). The blastn genome comparison was performed and visualized with Easyfig 2.2.5. The *attL* region in MMB-1 is marked with a brown rectangle. The red lines in some genes indicate expected values lower than 1E-10 in a Protein BLAST alignment of each sequence against phage VP882.

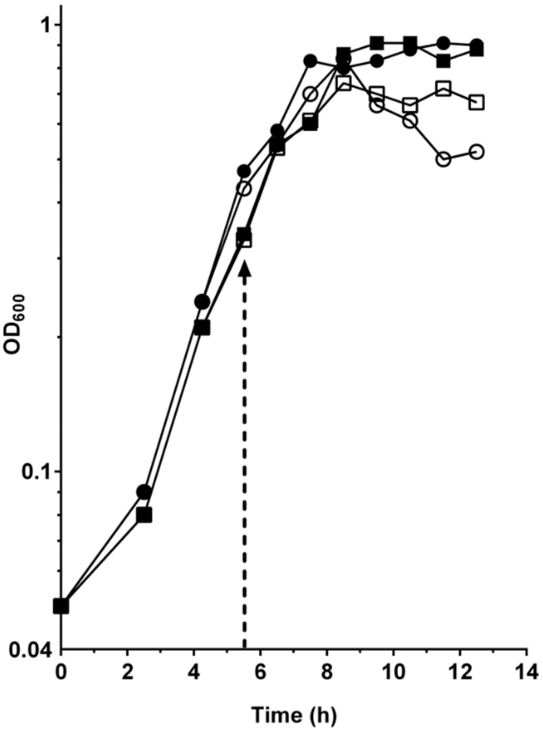

Figure S2. Induction of prophages by mitomycin C was not observed in the *M. mediterranea* strain with deletion of MEDPRO2. Evolution of OD600 for cultures of *M. mediterranea* strains MMB-1RSS (circles), and MMB-1RSS ΔMEDPRO2 (squares). Closed symbols represent control cultures, while open symbols represent cultures induced by the addition of mitomycin at a final concentration of 0,1 μg/ml at the time indicated by the arrow.
